# Supplementary material for: Rectus Femoris Cross-Sectional Area and Phase Angle asPredictors of 12-Month Mortality in Idiopathic Pulmonary Fibrosis Patients
Source: Nutrients. 2023 Oct 22;15(20):4473. doi: 10.3390/nu15204473 (PMC10609753; doi:10.3390/nu15204473)
Supplement: Supplementary file 1 [file nutrients-15-04473-s001.zip › nutrients-2643701-supplementary.pdf]

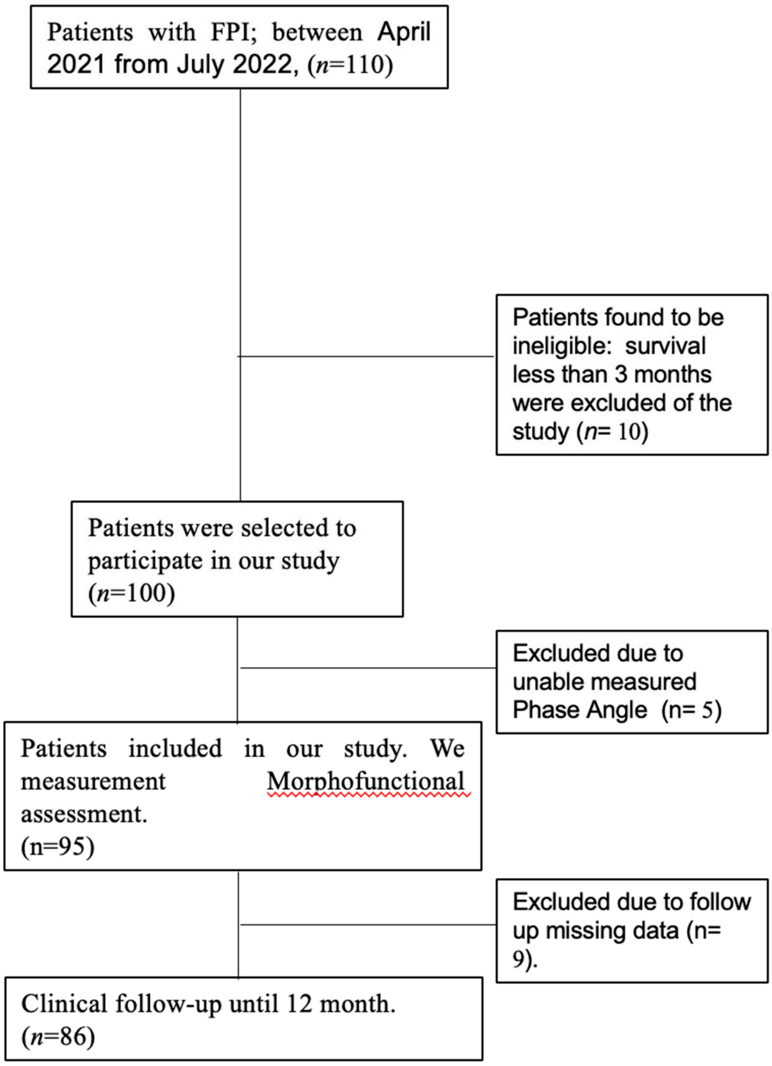

Figure S1. Flow chart in idiopathic pulmonary fibrosis patients.

Table S1. Baseline characteristics of the population of study divided by sex

	**All** *N=86*	**Males** *N=71*	**Females** *N=15*	***p* value**
**Demographic variables**				
Age (years)	71.0 (7.26)	71.0 (6.88)	71.1 (9.11)	0.969
Weight (kg)	78.30 (12.1)	79.4 (12.0)	73.4 (12.0)	0.083
Weight loss (%)	6.41 (6.78)	6.79 (6.90)	4.62 (6.09)	0.263
BMI (kg/m^2)	27.40 (3.44)	27.1 (3.21)	28.9 (4.15)	0.063
**BIA**				
PA (º)	4.78 (0.77)	4.85 (0.80)	4.49 (0.52)	0.101
SPA	-1.03 (1.01)	-1.08 (1.00)	-0.763 (1.03)	0.267
Hidratation (%)	74.60(2.31)	74.7 (2.41)	74.2 (1.81)	0.412
NaK	1.18 (0.18)	1.19 (0.18)	1.12 (0.14)	0.198
BCM (kg)	**25.5 (5.17)**	**26.6 (4.86)**	**20.3 (2.87)**	**<0.001***
FFMI (%)	**19.0 (1.74)**	**19.3 (1.71)**	**17.8 (1.32)**	**<0.001***
FM (kg)	**23.8 (7.91)**	**22.9 (7.22)**	**28.3 (9.65)**	**0.014 ***
SMI (cm^2/m^2)	**8.81 (1.20)**	**9.16 (0.95)**	**7.14 (0.78)**	**<0.001***
**Echography exploration**				
RF-CSA (cm^2)	**3.38 (0.98)**	**3.57 (0.96)**	**2.49 (0.45)**	**<0.001***
RF-CIR (cm)	**8.15 (1.11)**	**8.43 (0.96)**	**6.86 (0.78)**	**<0.001***
RF-X axis (cm)	**3.43 (0.50)**	**3.55 (0.44)**	**2.91 (0.41)**	**<0.001***
RF-Y axis (cm)	**1.11 (0.27)**	**1.14 (0.28)**	**0.98 (0.16)**	**0.046***
L-SAT (cm)	**0.78 (0.52)**	**0.53 (0.26)**	**0.86 (0.33)**	**<0.001***
RF-CON(cm)	1.57 (0.33)	1.59 (0.36)	1.49 (0.09)	0.601
T-SAT (cm^2)	**1.67 (0.71)**	**1.54 (0.64)**	**2.26 (0.75)**	**<0.001***
S-SAT (cm^2)	**0.72 (0.30)**	**0.65 (0.24)**	**1.05 (0.33)**	**<0.001***
VAT (cm^2)	0.65 (0.30)	0.625 (0.26)	0.76 (0.43)	0.106
**Functional measurement**				
HGS max (kg)	**34.5 (10.4)**	**37.5 (8.62)**	**20.7 (5.44)**	**<0.001***
HGS mean (kg)	**33.0 (10.1)**	**36 (8.31)**	**19.2 (4.70)**	**<0.001***
TUG (s)	8.29 (5.24)	7.96 (5.31)	9.85 (4.72)	0.222
6MW (m)	405.0 (76.2)	410 (77.5)	375 (64.1)	0.263
**Biochemical variables**				
Glucose (mg/dL)	110 (38.3)	113 (39.4)	85.0 (4.58)	0.242
Urea (mg/dL)	**43.5 (14.8)**	**45.8 (12.4)**	**27.6 (23.3)**	**0.043***
Creatinine (mg/dL)	1.05 (0.25)	1.07 (0.25)	0.83 (0.07)	0.202
Total cholesterol (mg/dL)	198 (58.8)	192 (54.3)	199 (40.7)	0.064
Triglycerides (mg/dL)	142 (90.5)	142 (95.6)	142 (48.1)	0.996
HbA1c (%)	5.97 (1.18)	6.07 (1.21)	5.10 (0.79)	0.470
Proteins (g/dL)	6.90 (0.42)	6.98 (0.62)	6.96 (0.46)	0.673
Albumin (g/dL)	3.81 (0.35)	3.86 (0.36)	3.55 (0.21)	0.288
Pre-albumin (mg/dL)	19.6 (32.7)	22.5 (3.67)	29.0 (5.30)	0.096
CRP (mg/L)	37.0 (138.0)	38.9 (142)	0.00 (0.00)	0.791

	All	Males	Females	*p* value
	N=86	***N=71***	***N=15***	
Vit D (mg/dl)	23.3 (14.2)	21.0 (13.5)	19.0 (14.0)	0.223
FCV(%)	67.9 (15.9)	67.4 (14.8)	69.4 (21.6)	0.682
FEV1(%)	77.9 (19.4)	76.5 (17.7)	83.2 (25.3)	0.271
**Clinicopathological variables**				
Diagnostic (month)	15.5 (19.2)	32.2 (20.8)	24.2 (12.2)	0.746
GAP Stage:				**0.034***
I	26 (34.2%)	18.0 (23.7%)	8.0 (10.5%)	
II	36 (47.4%)	32.0 (42.1%)	4.0 (5.30%)	
III	14 (18.4%)	13.0 (17.1%)	1.0 (1.30%)	

Data are expressed as mean ± standard deviations or percentage. Groups were divided 12 months-mortality variable. Asterisk indicates significant difference between groups, according to Welch's two sample test (*p<0.05). Chi squared test was used for variables expressed as percentage (***p<0.001, **p<0.01, *p<0.05). Data are expressed as mean ± standard deviations or percentage. Groups were divided 12 months-mortality variable. Asterisk indicates significant difference between groups, according to Welch's two sample test (*p<0.05). Chi squared test was used for variables expressed as percentage (***p<0.001, **p<0.01, *p<0.05).
Abbreviations: BMI: Body mass index; BIVA: Bioelectrical Impedance Vectorial Analysis; Pha: phase angle; Spha: Standardized phase angle; NaK: sodium potassium index; BCM.: Body mass cell; FFM: Fat free mass; ASMM: Apendicular squeletal mass mucle; SMI: Squeletal mass index; ; FFMI: Fat free mass index; FM: Fat mass; HGS mean: mean hand grip strenght; HGS max: maximum hand grip strenght; TUG: Up & Go test; 6MWT (6 minuts walk test); RF- CIR: circumference of quadriceps rectus femoris; RF-CSA: rectus femoris cross-sectional area; RF-X Axis; rectus femoris X axis; RF-Y Axis: rectus femoris Y axis; L-SAT: leg subcutaneous adipose tissue; SAT: Abdominal subcutaneous adipose Tissue, total (T), superficial (S) and VAT: visceral adipose tissue; FCV: forced vital capacity; FEV1: forced expiratory volume in the first second; DLCO: Pulmonary carbon monoxide diffusing capacity; GAP: gender (G), age (A) and physiology (P) index; SGA: subjective global assestment.
